# Evaluation of Pirfenidone and Nintedanib in a Human Lung Model of Fibrogenesis

**DOI:** 10.3389/fphar.2021.679388

**Published:** 2021-10-12

**Authors:** KM Roach, E Castells, K Dixon, S Mason, G Elliott, H Marshall, MA Poblocka, S Macip, M Richardson, L Khalfaoui, P Bradding

**Affiliations:** ^1^ Department of Respiratory Sciences, Institute for Lung Health, University of Leicester, Leicester, United Kingdom; ^2^ Mechanisms of Cancer and Ageing Lab, Department of Molecular and Cell Biology, University of Leicester, Leicester, United Kingdom; ^3^ FoodLab, Faculty of Health Sciences, Universitat Oberta de Catalunya, Barcelona, Spain

**Keywords:** pirfenidone, nintedanib, senicapoc, fibrosis, lung, IPF, human model, K_Ca_3.1

## Abstract

**Introduction:** Idiopathic pulmonary fibrosis (IPF) is a progressive, fatal lung disease with a poor prognosis and increasing incidence. Pirfenidone and nintedanib are the only approved treatments for IPF but have limited efficacy and their mechanisms of action are poorly understood. Here we have examined the effects of pirfenidone and nintedanib in a human model of lung fibrogenesis, and compared these with the putative anti-fibrotic compounds Lipoxin A4 (LXA4), and senicapoc, a K_Ca_3.1 ion channel blocker.

**Methods:** Early fibrosis was induced in cultured human lung parenchyma using TGFβ1 for 7 days, ± pirfenidone, nintedanib, or LXA4. Pro-fibrotic responses were examined by RT-PCR, immunohistochemistry and soluble collagen secretion.

**Results:** Thirty six out of eighty four IPF and fibrosis-associated genes tested were significantly upregulated by TGFβ1 in human lung parenchyma with a ≥0.5 log2FC (*n* = 32). Nintedanib (*n* = 13) reduced the mRNA expression of 14 fibrosis-associated genes including MMPs (MMP1,−4,−13,−14), integrin α2, CXCR4 and PDGFB, but upregulated α-smooth muscle actin (αSMA). Pirfenidone only reduced mRNA expression for MMP3 and −13. Senicapoc (*n* = 11) previously attenuated the expression of 28 fibrosis-associated genes, including αSMA, several growth factors, collagen type III, and αV/β6 integrins. Pirfenidone and nintedanib significantly inhibited TGFβ1-induced fibroblast proliferation within the tissue, but unlike senicapoc, neither pirfenidone nor nintedanib prevented increases in tissue αSMA expression. LXA4 was ineffective.

**Conclusions:** Pirfenidone and nintedanib demonstrate modest anti-fibrotic effects and provide a benchmark for anti-fibrotic activity of new drugs in human lung tissue. Based on these data, we predict that the K_Ca_3.1 blocker senicapoc will show greater benefit than either of these licensed drugs in IPF.

## Introduction

Idiopathic pulmonary fibrosis (IPF) is a progressive lung disease with a median survival of only 3–5 years, worse than many common cancers (Navaratnam et al., 2011; Raghu et al., 2011). It is characterised by parenchymal lung fibrosis leading to impairment of gas exchange and death (King et al., 2011). Although there are recognised risk factors such as smoking, the aetiology of IPF remains unknown. Repetitive damage to the alveolar epithelium and capillary endothelium, activation and proliferation of myofibroblasts and the disordered deposition of extracellular matrix (ECM) result in destruction of the alveolar architecture. Transforming Growth Factor beta 1 (TGFβ1) released following epithelial cell damage is a key upstream pro-fibrotic growth factor driving IPF pathophysiology (Raghu et al., 1989; Border and Noble, 1994; Branton and Kopp, 1999; Leask and Abraham, 2004).

There are only two drugs approved for the treatment of IPF, nintedanib and pirfenidone. Both drugs slow disease progression, but only modestly, and are often poorly tolerated (King et al., 2014). There therefore remains a major unmet clinical need. Pirfenidone exerts anti-fibrotic, anti-oxidant and anti-inflammatory effects to reduce lung collagen synthesis and deposition in bleomycin animal models, and in animal and human lung fibroblasts (Hilberg et al., 2012; Lehtonen et al., 2016; Knüppel et al., 2017). However, the molecular mechanisms of pirfenidone have not been fully elucidated as most mechanistic studies have concentrated on its effects in isolated lung cells, such as fibroblasts (Conte et al., 2014; Epstein Shochet et al., 2018; Jin et al., 2019). Nintedanib, in comparison, is an inhibitor of the platelet-derived growth factor (PDGF), vascular endothelial growth factor (VEGF) and fibroblast growth factor (FGF) receptor tyrosine kinases, collectively inhibiting signalling pathways involved in proliferation, migration and maturation of lung fibroblasts (Hostettler et al., 2014). Its anti-fibrotic activity has been demonstrated in animal models, *in vitro* assays and in clinical trials (Richeldi et al., 2014; Wollin et al., 2014; Wollin et al., 2015; Knüppel et al., 2017). To date, the molecular effects of these drugs have not been tested extensively in human lung tissue where cell function can be studied in the natural 3D tissue environment. Understanding the mechanisms of action these drugs have in human lung tissue and their relative efficacy may uncover anti-fibrotic pathways that can be targeted more effectively, clarify where these drugs fail to influence the fibrotic process, as well as providing a benchmark for the assessment of novel putative anti-fibrotic compounds.

Several studies have confirmed that human lung tissue is a valuable tool for the study of early pro-fibrotic signalling pathways (Alsafadi et al., 2017; Lehmann et al., 2018; Roach et al., 2018). Perhaps more importantly, these models are capable of screening and validating novel anti-fibrotic therapies rapidly and effectively, and therefore have great potential as more accurate predictors of clinical drug efficacy than currently used animal models. Our recently described model uses TGFβ1 to stimulate early fibrotic events in *ex vivo* human lung tissue, and recapitulates many of the gene expression changes present in IPF tissue (DePianto et al., 2015; Roach et al., 2018). This model does not require lung inflation with agarose which is major technical advantage, and is sensitive to drug interventions (Roach et al., 2018).

To-date the only study of pirfenidone and nintedanib in human lung tissue investigated the effects of these drugs on alveolar epithelial cell gene expression in precision cut lung slices stimulated with a pro-fibrotic cocktail from three donors (Lehmann et al., 2018). We have therefore used our model to investigate the molecular and histological pathways impacted by pirfenidone and nintedanib, and have compared these with the putative anti-fibrotic compounds senicapoc, an inhibitor of K_Ca_3.1 ion channels (Roach and Bradding, 2020), and the resolving mediator lipoxin A4 (LXA_4_) (Roach et al., 2015a).

## Materials and Methods

### Tissue Collection

Human lung tissue samples (*n* = 32) were obtained from healthy areas of lung from patients undergoing lung resection for carcinoma at Glenfield Hospital, United Kingdom. All patients gave written informed consent and the study was approved by the National Research Ethics service (references 10/H0402/12, 07/MRE08/42 and 17/EM/0231). Samples obtained were anonymised and coded before use.

### Tissue Explant Culture Model

2 mm^3^ pieces of human lung tissue were generated as described previously (Roach et al., 2018). Tissue was cultured in DMEM + vehicle control (0.1% DMSO) ± TGFβ1 (10 ng/ml) as described (Roach et al., 2018), or DMEM + TGFβ1 (10 ng/ml) + clinically/biologically relevant concentrations of nintedanib (1 µM), pirfenidone (500 µM), or lipoxinA4 (10 nM) (Roach et al., 2015a; Ogura et al., 2015; Knüppel et al., 2017; Lehmann et al., 2018). Separate plates and tissue were used for RNA experiments and immunohistochemistry experiments. Supernatants were collected on day 4 and stored at −80°C, and fresh media containing the same compounds was added (LXA_4_ was also added daily). Tissue was collected on day 0, 4 and 7 for RNA extraction. Tissue was collected for immunohistochemistry on Day 7. A schematic of the model is depicted in supplementary. Supplementary Figure S1.

### RNA Extraction

RNA extraction was performed as described previously. Tissue was dissociated using Precellys® 24 tissue homogenizer (Bertin Technologies, Montigny-le-Bretonneux, France) and total RNA purified using the automated QIAcube with RNeasy Fibrosis Mini kit (Qiagen, CA, United States) according to the manufacturer’s instructions. The RNA integrity was assessed with the Bioanalyzer 2,100 system (Agilent, CA, United States), and RIN values > 8 accepted as suitable for PCR profiling. RNA concentrations were then measured using the Nanodrop 2000 (Labtech International, East Sussex, United Kingdom).

### RT^2^ Profiling PCR Fibrosis Array

CDNA was pre-amplified using the RT^2^ first strand cDNA kit, according to manufacturer’s instructions (SabBioscience, Qiagen). A RT^2^ profiler human fibrosis PCR array (PAHS-120ZA) was performed for quantitative PCR in the Strategene MX3000P system according to the manufacturer’s instructions. The PCR plate performance and quality control criteria are described (Roach et al., 2018). The average CT values of beta-2-microglobulin (β2M) and beta actin (β-actin) housekeeping genes were used as normalising controls. Results were calculated using the 2^−ΔΔCt^ method (Livak and Schmittgen, 2001). A minimum and maximum log_2_ fold change (FC) of +5 to −5 was applied.

### Real Time PCR Analysis

Quantitative Real-time PCR (qRT-PCR) was used to measure mRNA expression levels of ACT2A [α-smooth muscle actin (αSMA)], COL1A2 (collagen type I), COL3A1 (collagen type III), and FN1 (fibronectin), using the Quantstudio five Real-Time PCR machine (Applied Biosystems). ACT2A, COL1A2, COL3A1 primers are validated (primer efficiency confirmed to be between 90 and 105%), sequenced and have previously been published (Roach et al., 2014; Roach et al., 2015a; Roach et al., 2015b). Primers for FN1 (fibronectin) (Hs_FN1_1_SG, QT00038024) and housekeeping control B2M (beta-2-microglobulin) (Hs_B2M_1_SG, QT00088935) were quantitect primer assays acquired from Qiagen, Germany. Gene expression was quantified using Brilliant SYBR Green QRT-PCR 1-Step master mix (Strategene, Netherlands). All expression data were normalized to B2M and corrected using the reference dye ROX. PCR products were run on a 1.5% agarose gel to confirm product size and each product was sequenced to confirm specificity of the primers. Results were calculated using the 2^−ΔΔCt^ method.

For the analysis of senescence markers total RNA was extracted using ReliaPrep™ RNA Cell Miniprep (Promega, Z6011). RT-qPCR analysis was used to measure the levels of senescence markers using gene-specific primer pairs (Supplementary Table S1) and SYBR Green master mix (Invitrogen). The qPCR reactions were propagated on a LightCycler^®^ 480 system (Roche). Relative quantity values were obtained based on the comparative Ct method. Results were analysed in Microsoft Excel and graphs plotted using GraphPad Prism 7.0 Software.

### Paraffin Embedding and Immunohistochemistry

Tissue specimens were formalin fixed for 24 h at room temperature and paraffin-embedded. Tissue sections of 4 µm thickness were cut onto charged glass slides, de-waxed in xylene and rehydrated through graded industrial methylated spirits (IMS). Dependent on antibody optimization, antigen retrieval was performed using either high or low pH (see Supplementary Table S2). Immunostaining was performed using the EnVison Flex staining kit (Dako K8002), following the manufacturer’s instructions. A full list of the primary antibodies, concentrations and isotype control antibodies can be found in supplementary table 2. Colour development was performed using 3, 3′ diaminobenzidine tetrahydrochloride (DAB) substrate and sections were counter stained in Gills haematoxylin and mounted with Dibutylphthalate Polystyrene Xylene (DPX). Digital pathology was performed as previously described (Roach et al., 2018), using both Zen by Zeiss and QuPath software. Four sections, one from each of four pieces of tissue, were analysed, and the mean measurement from the four sections was calculated. Fibroblast-specific protein-positive (FSP+) cells and αSMA + cells were expressed as the percentage of total cells. Only FSP + cells that were fibroblast-like were counted; FSP also stains macrophages which were excluded from the counts based on their cell size and obvious characteristics. Collagen type 1 expression was expressed as the mean intensity of staining as it was widespread and diffuse, while collagen type 3 expression was expressed as the percentage area stained because it was patchy in its distribution. This work was performed in a immunohistology laboratory that is GCP/GLP and ISO9001-2015 compliant.

### Statistics

Experiments from an individual donor were performed in duplicate or triplicate and a mean value was derived for each condition. The RT^2^ profiling gene array has in-built reproducibility controls and therefore only one plate per donor was run.

Data distribution across donors was tested for normality using the Kolmogorov-Smirnov test. For parametric data, the one-way ANOVA or repeated measures ANOVA for across group comparisons was used followed by the appropriate multiple comparison post hoc test; A paired *t* test was used for groups of only two conditions on the same donor. For non-parametric data, the Friedman test was used for across group comparison of paired data followed by the appropriate multiple comparison post hoc test; otherwise a Wilcoxon rank sum test was used. Graph Pad Prism (version 6, GraphPad Software, San Diego, CA, United States) was used for these analyses. A value of *p* < 0.05 was taken to assume statistical significance and data are represented as mean (±SEM) or median (±IQR).

False discovery analysis was performed on all RT^2^ PCR profiling array data to overcome the problems of multiple testing. This was undertaken using John D. Storey adjusted *p*-value <0.05 and q-value of 0.05 (5% FDR) derived from the full list of *p*-values. The factor analysis was carried out in SPSS on the fold change data of the TGFβ1 derived expression as described (Roach et al., 2018).

## Results

Assessment of tissue viability, cellular senescence, gene expression and protein expression in human lung tissue exposed to TGFβ1.

**FIGURE 1:**
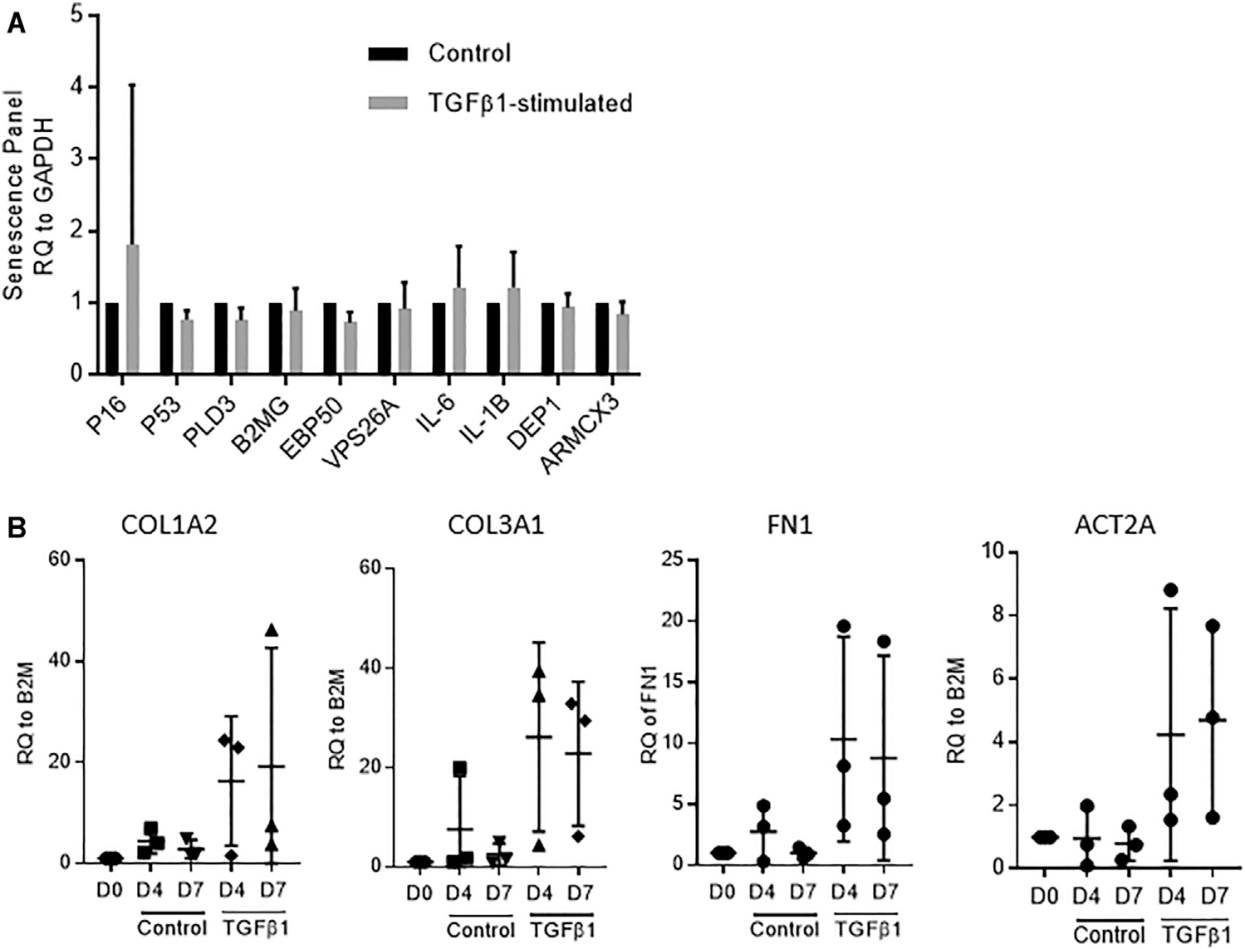
**(A)** No evidence of cellular senescence was detected by RT-PCR in TGFβ1-stimulated tissue compared to matched controls using a panel of 10 diverse senescence markers (*n* = 5 donors). **(B)** A selection of fibrotic markers, COL1A2, COL3A, FN1 and ACT2A quantified by RT-PCR, showed early fibrotic changes in human TGFβ1-stimulated lung tissue as early as day 4, *n* = 3.

We have previously demonstrated that control and TGFβ1-stimulated human lung tissue is viable over 7 days with no evidence of nuclear fragmentation, tissue necrosis or reduction in metabolic activity (MTS turnover), and that RNA integrity is maintained (Roach et al., 2018). This was mirrored in the current experiments, and RNA integrity was again preserved with all RIN values > 8. To examine whether culture of the tissue in TGFβ1 promoted cellular senescence we examined senescence markers via RT-PCR. We found no difference between control and TGFβ1-treated tissue after 7 days, using a panel of diverse senescence markers, including p16, p21, p53 (Herranz and Gil, 2018) together with markers of the senescence secretome IL-1β, IL-6 (McHugh and Gil, 2018) and recently identified membrane-specific proteins preferentially expressed in senescence cells (Althubiti et al., 2014) (*n* = 5 donors) (Figure 1A).

Early fibrotic changes were present in the TGFβ1-stimulated tissue at both day 4 and day 7 identified using RT-PCR on a select panel of key fibrosis-associated genes, including the myofibroblast activation marker αSMA (ACT2A), and extracellular matrix deposition markers fibronectin (FN1), collagen type 1 (COL1A2) and collagen type 3 (COL3A1) (*n* = 3) (Figure 1B).

**FIGURE 2:**
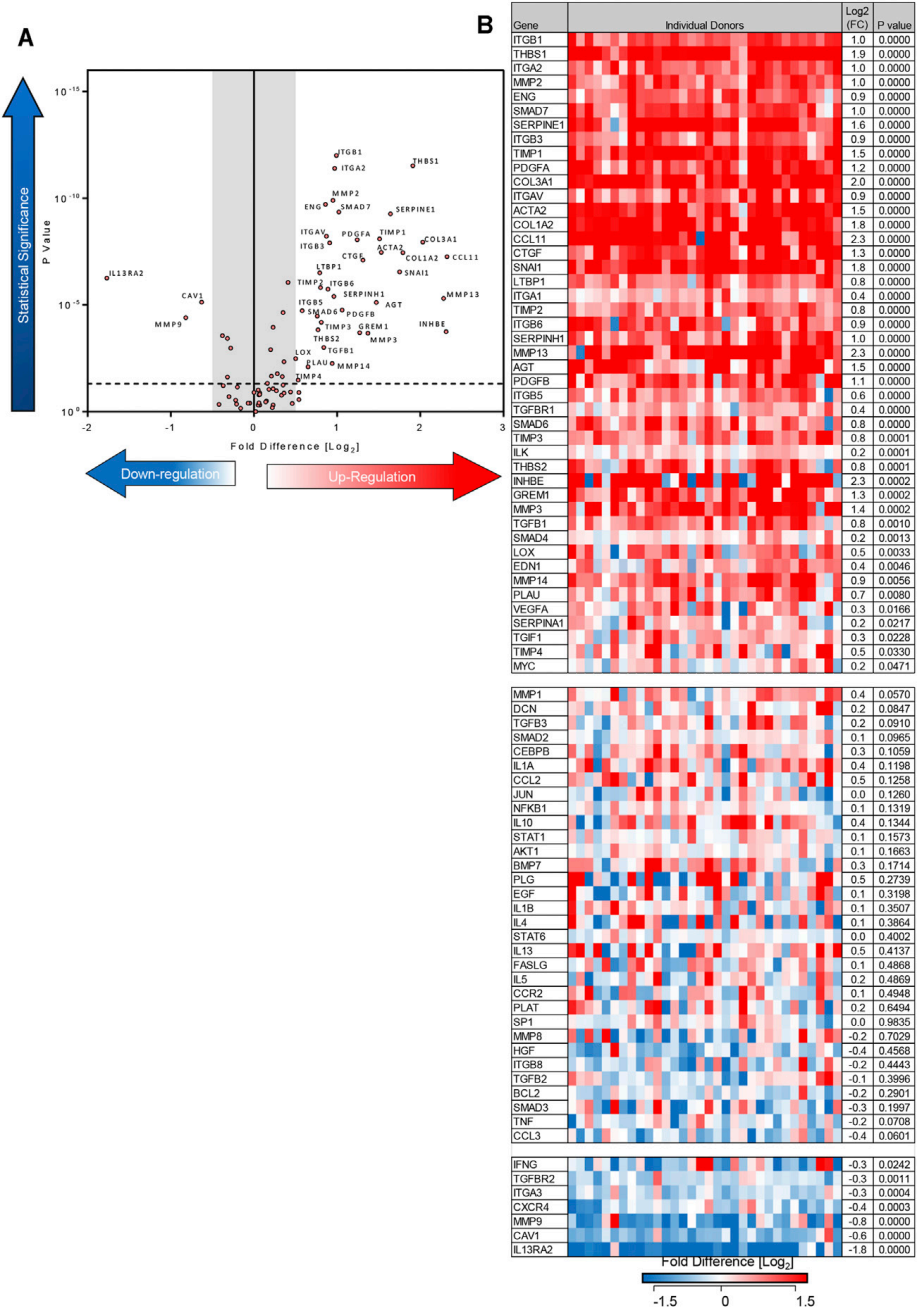
Differentially expressed genes in human lung parenchyma after 7 days stimulation with TGFβ1 compared to control tissue. **(A)** A volcano plot identifying the statistically significant genes regulated by TGFβ1 shown as *p* value versus log_2_FC. Out of 84 genes, 36 were significantly upregulated and three downregulated following a 5% false discovery analysis with John D. Storey adjusted *p* value <0.05 and absolute value of log_2_FC ≥ 0.5, depicted by the dotted lines and grey shaded area (results are mean of 32 individual donors). **(B)** The log_2_FC of all 84 genes in individual donors is depicted in this heatmap, with mean log_2_FC, and statistical significance indicated. Significance was calculated using a student’s t-test on the ΔΔCT for each gene.

**FIGURE 3:**
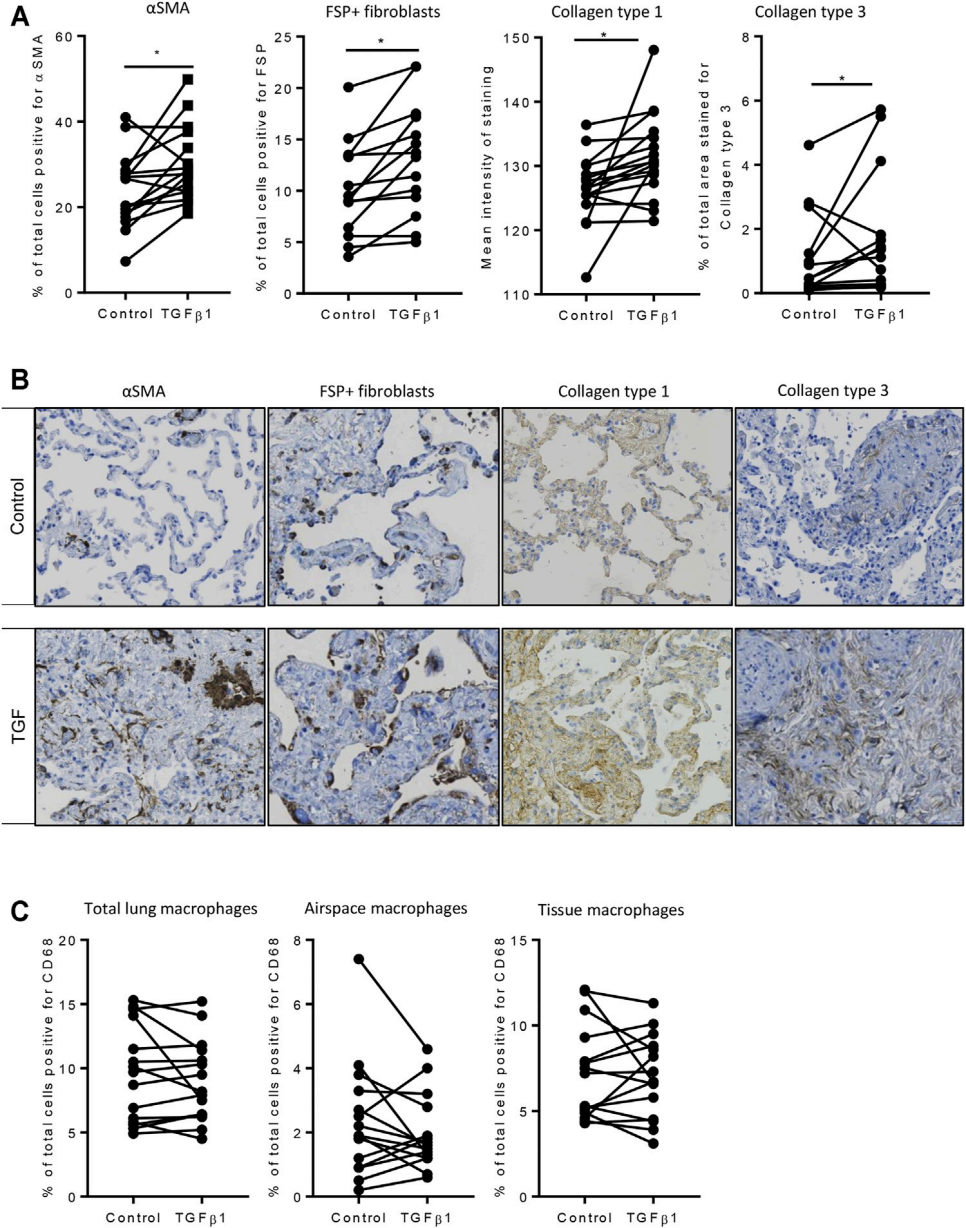
Immunohistochemical analysis of TGFβ1-stimulated tissue compared to matched control tissue. **(A**) Immunostaining was significantly increased for αSMA (*p* = 0.0093, *n* = 17), FSP + fibroblast-like cells (*p* = 0.0025, *n* = 14), in TGFβ1 (10 ng/ml)-stimulated tissue compared control tissue following 7 days of *ex vivo* culture, collagen type 1 (*p* = 0.0121, *n* = 17), and collagen type 3 (*p* = 0.0302, *n* = 15). *p < 0.05 paired *t* test. **(B)** Representative immunohistochemical staining is depicted for each antibody for both control and TGFβ1-stimulated tissue following 7 days of *ex vivo* culture. Relevant isotype controls were negative (images not shown). **(C)** The percentage of CD68 ^+^ macrophages showed no significant changes in total, airspace or resident tissue macrophages numbers between control and TGFβ1-exposed lung tissue (*n* = 15).

Using the 84-well RT^2^ fibrosis array, at day 7 there was upregulation of fibrosis-associated gene expression as seen previously (Roach et al., 2018). In keeping with our previous work, 45 out of 84 pro-fibrotic and pro-inflammatory gene transcript levels associated with IPF were increased significantly in the TGFβ1-treated tissue and 36/45 of these demonstrated a biologically relevant ≥0.5 log_2_FC increase when compared to control (*n* = 32 donors). Genes that were upregulated encoded tissue remodelling growth factors (CTGF, PDGFA and B, TGFβ1), cell adhesion molecules (integrins αv, β1, β6), ECM remodelling enzymes (matrix metalloproteinases [MMPs] 2,-3,-13,-14), extracellular matrix constituents (collagens type I and 3), transcription factors (SMAD6,-7) and αSMA (Figures 2A,B **)**. Immunohistochemical analysis showed that following TGFβ1 treatment there were quantifiable increases in lung parenchymal collagen type 1 (*n* = 17) and 3 (*n* = 15), and αSMA+ (*n* = 17) and FSP + cells (*n* = 14) (Figures 3A,B). The PCR and immunohistochemistry results are consistent with our previously published work (Roach et al., 2018) and observations in IPF tissue (DePianto et al., 2015; Zhang et al., 2019).

In addition to upregulating mRNA for pro-fibrotic molecules, TGFβ1 also downregulated mRNA for three molecules with a ≥0.5 log_2_FC (IL13RA2, MMP9, caveolin 1). Downregulation of these molecules potentially promotes fibrosis (Cabrera et al., 2007; Lumsden et al., 2015; Marudamuthu et al., 2019). Diagrams summarising the fibrotic pathways regulated by TGFβ1 exposure in this model are summarised in Supplementary Figure S2.

Resident tissue macrophages are key regulators of tissue repair and fibrosis, and disturbances in their function have been associated with IPF (Wynn and Vannella, 2016; Misharin et al., 2017). We found no change in total, airspace or tissue macrophages as a percentage of total cells between control and TGFβ1-exposed lung tissue (Figure 3C).

### Repeatability and Reproducibility of the Human Model of Lung Fibrosis

**FIGURE 4:**
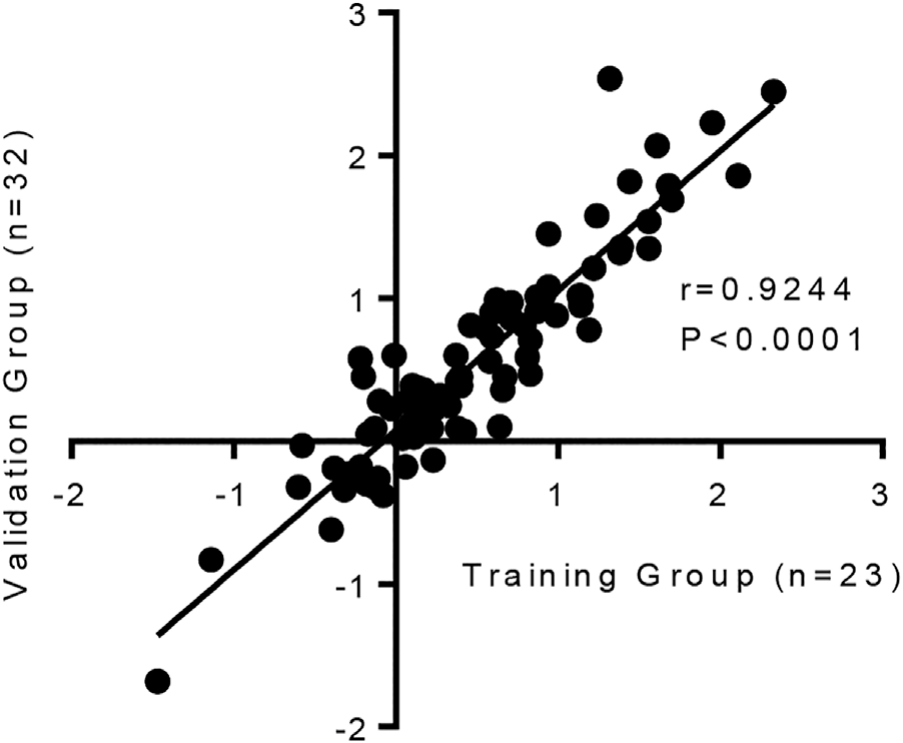
The human lung response to TGFβ1 exposure is consistent, with a strong correlation in the changes of mRNA expression for 84 fibrosis-associated genes between our previous published work (*n* = 23 human lung donors) (Roach et al., 2018) and the subsequent *n* = 32 human samples we have tested in this study (*p* < 0.0001, r = 0.9244).

It is important to show that this model is reproducible. Changes in the 84 fibrosis-associated genes following TGFβ1 treatment were strongly correlated between our original published work (*n* = 23 human lung donors), and the subsequent *n* = 32 human donors used in this study (Figure 4).

Collectively these results highlight that even though there is known heterogeneity in human tissue responses, which are evident between donors here, overall the human lung response to repeated TGFβ1 exposure is consistent.

### The Effects of Pirfenidone and Nintedanib on Pro-fibrotic Gene Expression in TGFβ1-Stimualated Human Lung Tissue

**FIGURE 5:**
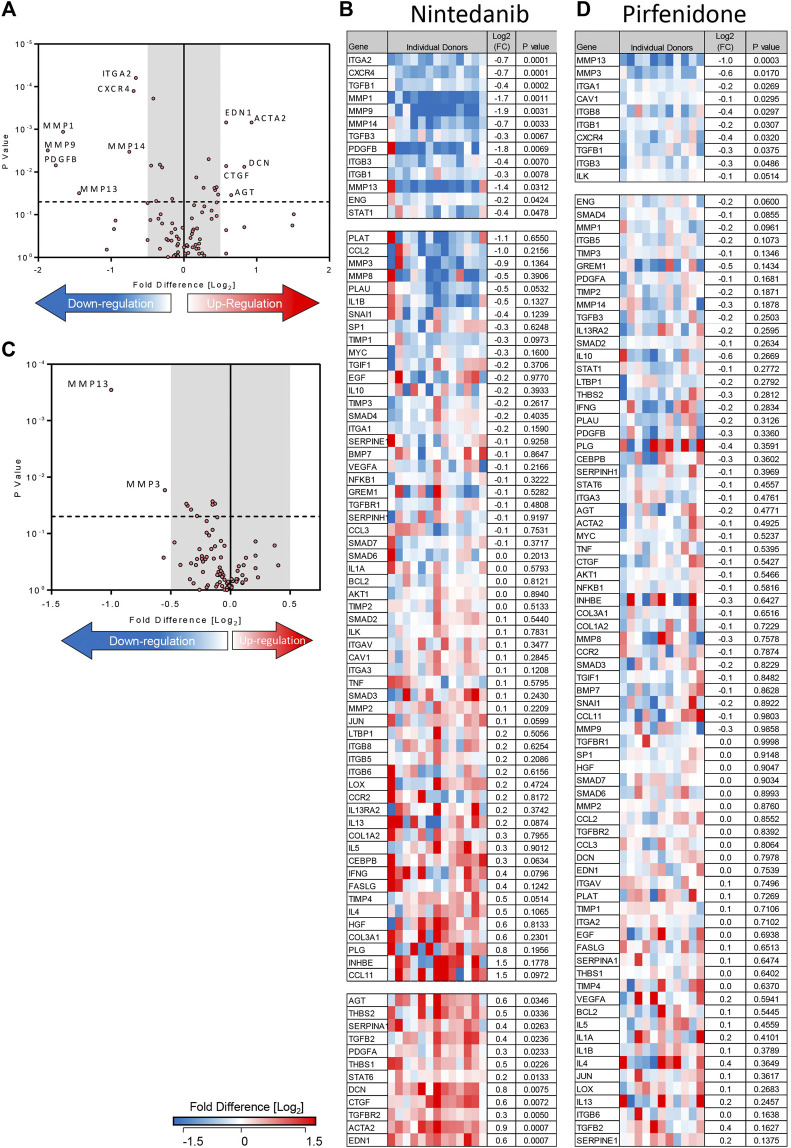
Nintedanib and pirfenidone modulation of TGFβ1-dependent fibrotic gene expression in *ex vivo* human lung parenchyma. **(A**) A volcano plot demonstrating that out of 84 genes studied, mRNA for seven was significantly downregulated following treatment with nintedanib (1 µM) for 7 days in comparison to TGFβ1 stimulation alone, and seven genes were significantly upregulated (results are the mean of 13 individual donors). **(B**) The log_2_FC of all 84 genes in individual donors following treatment with nintedanib for 7 days in comparison to TGFβ1 stimulation alone is depicted in this heatmap. Mean log_2_FC and statistical significance are indicated. **(C)** A volcano plot demonstrating that out of 84 genes studied, two were significantly downregulated following treatment with pirfenidone (500 µM) for 7 days in comparison to TGFβ1-stimulation alone (results are mean of 11 individual donors). **(D)** The log2 fold regulation of all 84 genes in individual donors following treatment with pirfenidone for 7 days in comparison to TGFβ1 stimulation alone is depicted in this heatmap. Mean log_2_FC and statistical significance are indicated.

Treatment of TGFβ1-stimulated tissue with nintedanib (1 µM) inhibited the TGFβ1-dependent upregulation of 13 genes and of these, 7 were reduced with a ≥0.5 log_2_FC which is considered biologically relevant (*n* = 13 donors) (Figures 5A,B). Compared to TGFβ1 alone, the addition of nintedanib reduced ECM remodelling enzymes (MMP’s), cell adhesion molecules (ITGs), the growth factor (PDGF), and the chemokine receptor CXCR4 (Figures 5A,B). In comparison, pirfenidone (500 µM) only inhibited the upregulation of two genes (MMP3 and 13) with a ≥0.5 log_2_FC (*n* = 11 donors) (Figures 5C,D).

Although nintedanib attenuated many genes associated with IPF that were upregulated by TGFβ1, nintedanib also significantly increased the expression of seven genes with a ≥0.5 log_2_FC. Of these, decorin mRNA expression was not increased by TGFβ1 alone, but was upregulated by nintedanib, another potentially anti-fibrotic outcome (Giri et al., 1997). However six pro-fibrotic genes upregulated by TGFβ1, including genes encoding growth factors (CTGF), thrombospondin 1 and 2, angiotensinogen, and α-SMA, were further increased by nintedanib (Figures 5A,B). This was not evident with pirfenidone. The molecular pathways regulated by TGFβ1 and their modulation by nintedanib, pirfenidone and senicapoc are summarised in Supplementary Figures S3, S4.

### The Effects of Pirfenidone and Nintedanib on Protein Expression in TGFβ1-Stimulated Human Lung Tissue

**FIGURE 6:**
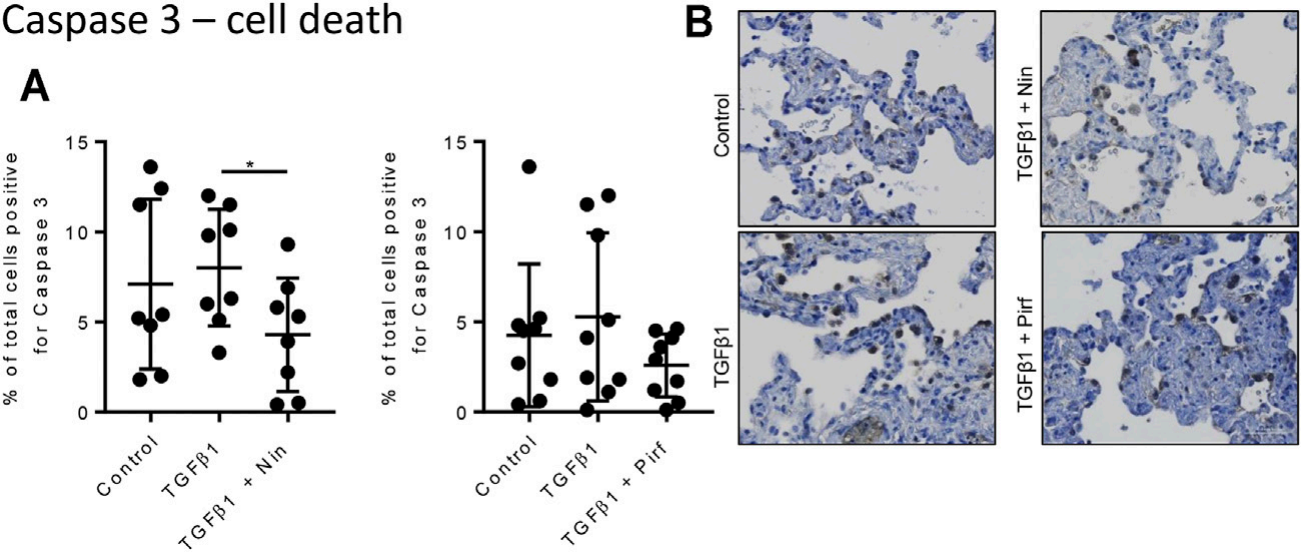
Immunohistological analysis of apoptotic cells assessed by caspase three staining. **(A)** The percentage cells positive for caspase three in human lung tissue following 7 days incubation with TGFβ1 ± pirfenidone or TGFβ1 ± nintedanib did not change compared to TGFβ1 alone. Nintedanib significantly reduced the number of caspase three positive cells (*p = 0.0021, paired *t* test). **(B)** Representative immunohistochemical caspase three staining following 7 days of *ex vivo* culture. Relevant isotype controls were negative (images not shown).

**FIGURE 7:**
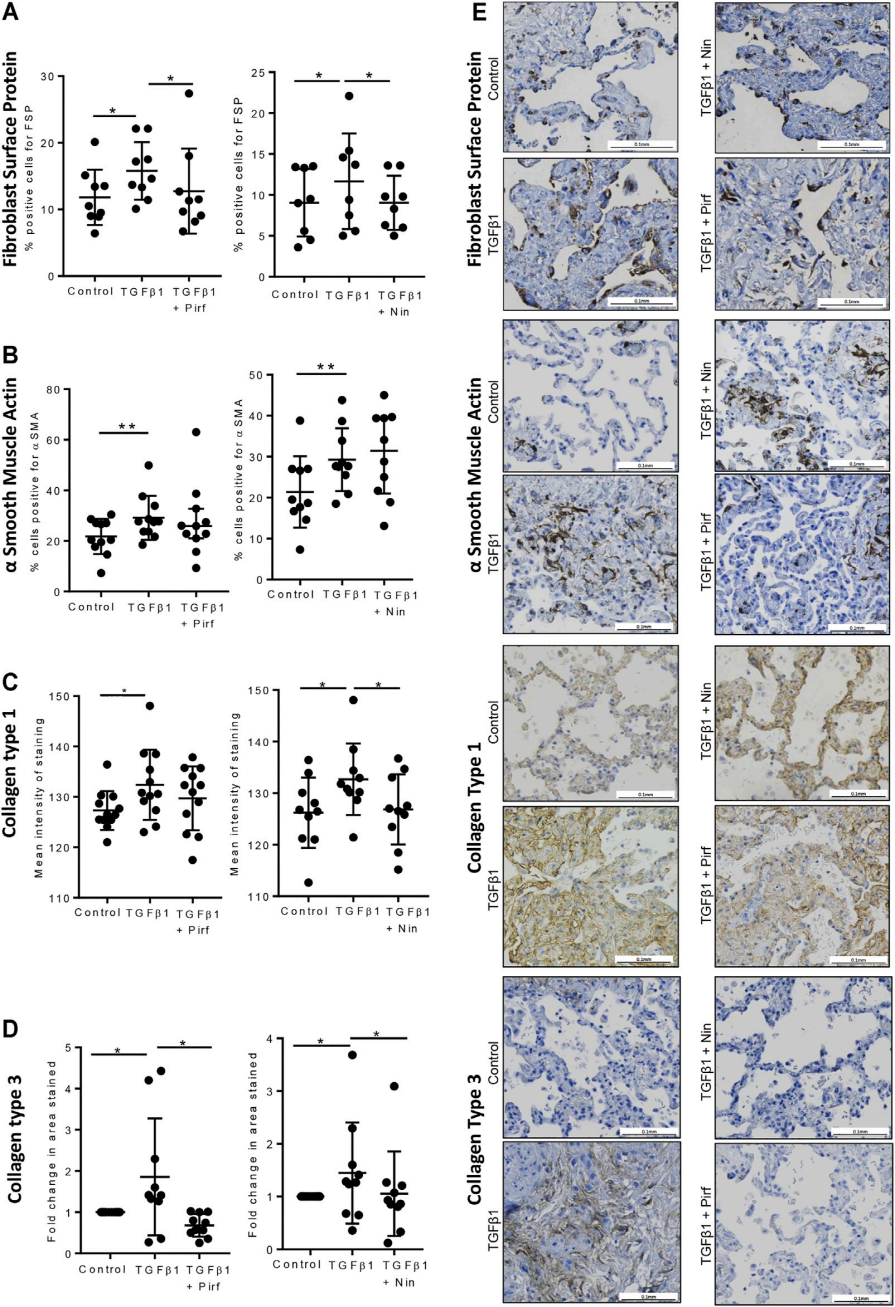
Immunohistochemical staining for FSP + fibroblasts, αSMA, collagen type 1, and collagen type 3, following lung tissue exposure to TGFβ1, with or without nintedanib (1 µM) or pirfenidone (500 µM) for 7 days. **(A**–**D**) Quantification of immunohistochemical staining. Nintedanib significantly attenuated FSP (*n* = 8), collagen type 1 (*n* = 10) and collagen type III (*n* = 10) TGFβ1-dependent pro-fibrotic responses within the tissue over 7 days *ex vivo* culture. Pirfenidone significantly attenuated FSP (*n* = 9) and collagen type III (*n* = 10) TGFβ1-dependent pro-fibrotic responses within the tissue over 7 days *ex vivo* culture. Data are presented as mean ± SEM *p < 0.05, **p < 0.01. **(E**) Representative immuno-histochemical images of 7 days *ex vivo* cultured lung tissue under the following conditions: un-stimulated control, TGFβ1-stimulated + 0.1% DMSO, TGFβ1-stimulated + nintedanib (1 µM) and TGFβ1-stimulated + pirfenidone (500 µM).

**FIGURE 8:**
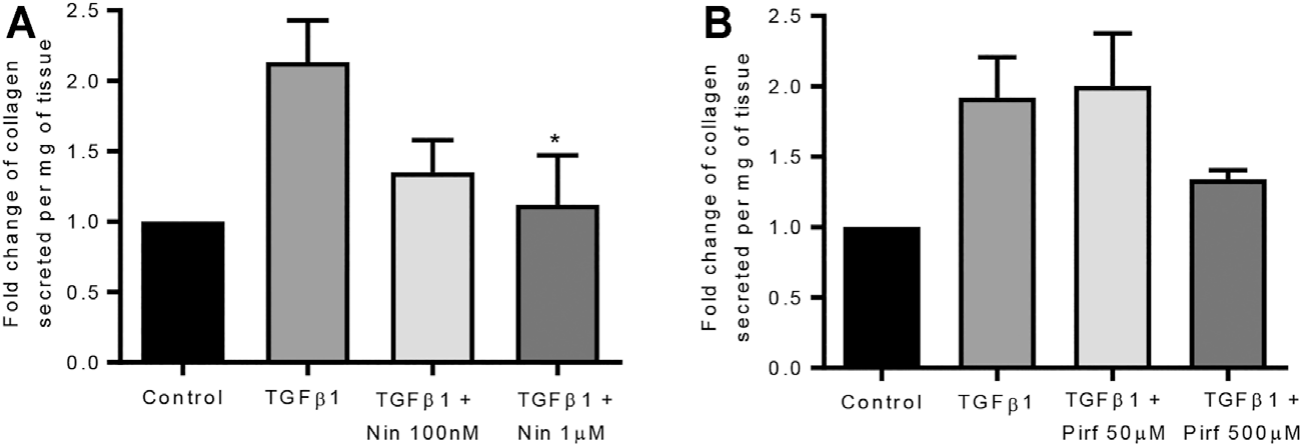
The effects of nintedanib and pirfenidone on soluble collagen secretion. **(A)** Nintedanib inhibited TGFβ1-dependent soluble collagen secretion into the supernatants following 4 days of *ex vivo* culture (*n* = 5, *p* = 0.040) (Data are presented as mean ± SEM *p < 0.05, paired *t* test). **(B)** Pirfenidone did not significantly attenuate soluble collagen secretion into the supernatants following 4 days of *ex vivo* culture (*n* = 5). The amount of collagen secretion was examined per mg of tissue present in the well.

Treatment of TGFβ1-stimulated tissue with nintedanib or pirfenidone did not have any pro-apoptotic effect on the cells within the tissue as measured by caspase three protein expression using immunohistochemistry (Figure 6). However, nintedanib significantly reduced the number of caspase three positive cells. Both nintedanib and pirfenidone reduced the number of fibroblasts within the tissue after 7 days of treatment with TGFβ1 as assessed by immunostaining for fibroblast-specific protein (FSP) (Figure 7A). This is in accordance with numerous studies which have shown pirfenidone and nintedanib inhibit isolated primary human lung fibroblast proliferation (Lehtonen et al., 2016; Conte et al., 2014). Neither pirfenidone nor nintedanib reduced the number of cells positive for αSMA (Figure 7B). Nintedanib inhibited both collagen type 1 and collagen type 3 expression in the TGFβ1-stimulated-tissue, whereas pirfenidone inhibited only collagen type 3 expression (Figures 7C,D). In addition to the collagen expressed within human lung tissue, we also assessed the quantity of collagen secreted into the supernatant over 4 days, using the Sircol assay. As previously reported, TGFβ1-stimulated tissue secreted significantly more collagen into the supernatant in comparison to control tissue, and both pirfenidone and nintedanib at clinically relevant concentrations (500 and 1 µM respectively) reduced this, although only nintedanib reached significance (Figures 8A,B). Neither pirfenidone nor nintedanib had any effect on the number of alveolar macrophages in keeping with Zhang et al., 2019 **(** Supplementary Figure S6 **)** (Zhang et al., 2019)**.**

### The Effect of Lipoxin A4 on TGFβ1-Induced Fibrogenesis

Lipoxin A4 inhibits TGFβ1-dependent HLMF pro-fibrotic activity *in vitro* (Roach et al., 2015a). Initially LXA_4_ was added at day 0 and day 4. Analysis of the first five experiments using the Qiagen RT2 PCR array showed no effect on TGFβ1-dependent effects (not shown). As LXA_4_ has a short half-life in tissues, we then tried daily dosing, replacing the medium daily. However, there was still no effect on TGFβ1-dependent changes mRNA expression (Supplementary Figure S7).

### Comparison of Nintedanib and Pirfenidone With the K_Ca_3.1 Ion Channel Blocker, Senicapoc

K_Ca_3.1 ion channels appear to play a key role in pathological fibrosis in many organs including the lung (Roach and Bradding, 2020). In our previous study describing this human lung model, the K_Ca_3.1 blocker senicapoc inhibited the TGFβ1-dependent upregulation of 28 fibrosis-associated genes with considerable effects on fibrosis-associated growth factors, members of the TGFβ1 superfamily, cell adhesion molecules, and transcription factors (Supplementary Figure S5) (Roach et al., 2018). Senicapoc did not upregulate any pro-fibrotic genes. Factor analysis demonstrated three sets of genes (factors) that appeared to be co-regulated following TGFβ1 exposure, and senicapoc was active in two of these factors (Table 1). Similarly, nintedanib was active in the same two factors but upregulated the expression of three pro-fibrotic genes and attenuated the expression of two genes with a ≥0.5 log_2_FC (Table 1). Pirfenidone, which only reduced the expression of two genes with a ≥0.5 log_2_FC, did not inhibit any of the genes in the three TGFβ1-dependent factors. The effects of all the drugs trialled to-date in this TGFβ1-dependent human lung model of fibrogenesis, (senicapoc, nintedanib, pirfenidone, dexamethasone and LXA_4_) are shown in Table 1, including data from previously published work (Roach et al., 2018).

**TABLE 1:** Factor analysis of TGFβ1-dependent human lung fibrogenesis, and the effects of drug interventions.

				Up/Downregualted log2FC ≥ 0.5□				
Genes	Factor 1	Factor 2	Factor 3	Senicapoc (n = 11)	Nintedanib (n = 13)	Pirfenidone (n = 11)	Dexamethasone (n = 5)	LipoxinA4 (n = 5)
TGFBR1	0.977	—	—	↓	—	—	—	—
SMAD4	0.968	—	—	↓	—	—	—	—
SMAD7	0.913	—	—	↓	—	—	—	—
TIMP2	0.876	—	—	—	—	—
PLAU	0.876	—	—	—	—	—	↓	—
SNAI1	0.865	—	—	—	—	—	—	—
TGFBR2	0.840	—	—	—	—	—	↑	—
ITGB1	0.813	—	—	—	↓
ITGA1	0.766	—	—	↓	—	—	—	—
THBS1	0.758	—	—	—	↑
TIMP1	0.745	—	
ITGB3	0.724	—	—	↓
THBS2	0.722	—	—	—	—	—	—	—
CXCR4	0.712	—	—	↓	↓
LOX	—	0.748	—	—	—	—	—	—
ACTA2	—	0.885	—	↓	↑	—	—	—
CAV1	—	0.944	—	↓	—	—	↑	—
COL1A2	—	0.979	
CTGF	—	0.915	—	—	↑	—	—	—
ENG	—	0.866	—	↓	↓	—	—
CCL2	—	0.848	—	—	—	—	—	—
ITGB5	—	0.927	—
CCL11	—	0.782	—	—	—	—	—	—
TIMP3	—	—	0.874
TIMP4	—	—	0.911	—	—	—	—	—
SERPINE1	—	—	0.780

In terms of protein changes in the tissue, and culture supernatant, senicapoc reduced tissue FSP+ and αSMA + cells, in keeping with it’s marked inhibitory effects on human lung myofibroblast differentiation (Roach et al., 2013; Roach et al., 2014; Roach et al., 2015b), whereas pirfenidone and nintedanib only inhibited the TGFβ1-dependent increase in FSP + cells. Overall, the anti-fibrotic effects of senicapoc appear more extensive than those of nintedanib and pirfenidone (Roach et al., 2018).

## Discussion

We have used our human tissue model of early lung fibrosis to perform the first detailed analysis of the anti-fibrotic effects of pirfenidone and nintedanib in human lung tissue stimulated with the key pro-fibrotic growth factor TGFβ1. Our data show that this model is highly reproducible in terms of its response to TGFβ1, and replicates many of the molecular changes characteristic of IPF such as increases in pro-fibrotic gene expression, tissue and soluble collagens, and increased myofibroblast numbers (DePianto et al., 2015). In total, the mRNA for 36 out of 84 fibrosis-associated genes was upregulated with a significant ≥0.5 log_2_FC in response to TGFβ1, representing ECM remodelling enzymes, ECM components, integrins, growth factors and TGFβ1 signalling components. A further three potentially anti-fibrotic genes were also downregulated by TGFβ1 with a significant ≥0.5 log_2_FC. Similar to IPF, we did not see a strong or persistent response of inflammatory cytokines and chemokines. In parallel, there were increases in fibroblasts expressing fibroblast-specific protein, cells expressing αSMA, deposition of type 1 and 3 collagens, and secretion of soluble collagen. This model therefore replicates several fibrotic processes and gene expression changes present in human IPF tissue (DePianto et al., 2015), and is reproducible.

The effects of both pirfenidone and nintedanib have been tested extensively in animal models of fibrosis and primary IPF-derived cells. Pirfenidone inhibits fibrosis in bleomycin-treated animals and inhibits human lung fibroblast proliferation, differentiation and collagen secretion through the inhibition of TGFβ1-dependent signalling (Hilberg et al., 2012; Lehtonen et al., 2016; Knüppel et al., 2017; Epstein Shochet et al., 2018; Jin et al., 2019). However, it’s precise molecular mode of action remains elusive. Nintedanib is an inhibitor of PDGF, VEGF and FGF receptor tyrosine kinases, and similar to pirfenidone, inhibits lung fibrosis in animal models and inhibits pro-fibrotic human lung fibroblast activity (Hostettler et al., 2014; Wollin et al., 2015; Lehtonen et al., 2016; Knüppel et al., 2017; Epstein Shochet et al., 2018). However, to-date only one study has reported the effects of pirfenidone and nintedanib in *n* = 3 human lung samples using a precision cut lung slice (PCLS) model that uses agarose to inflate whole lobes and uses a cocktail of TGFβ1, PDGF-AB, TNFα and lysophophatidic acid (Lehmann et al., 2018). This study focused on alveolar epithelial cell function, and showed that pirfenidone and nintedanib did not alter tissue viability, and that nintedanib treatment restored epithelial gene expression, prosurfactant protein C protein expression, and surfactant protein secretion that was suppressed by the pro-fibrotic cocktail (Lehmann et al., 2018). Pirfenidone did not have an impact on any of these markers of alveolar epithelial cell function. These data were supported by experiments in mouse bleomycin model lung slices and mouse type II alveolar epithelial cells.

Using our model of TGFβ1-dependent fibrogenesis, neither pirfenidone nor nintedanib inhibited type 1 or 3 collagen gene expression, although both drugs had significant inhibitory effects on interstitial type 1 and 3 collagen deposition as assessed by immunohistochemistry, and soluble collagen secretion, raising the possibility that they inhibit collagen production/secretion in human lung tissue through post translational effects. Nintedanib inhibited the gene expression for the chemokine receptor CXCR4 (implicated in cell recruitment and migration), the ECM remodelling enzymes MMP1, −9, −13 and −14, PDGFB, and the integrin α2. In addition, nintedanib increased the expression of decorin, a glycosaminoglycan that may inhibit TGFβ1 activity (Giri et al., 1997). Thus nintedanib clearly displays anti-fibrotic activity in this model. However, nintedanib also enhanced the TGFβ1-dependent expression of several profibrotic molecules (CTGF, thrombospondin 1 and 2, angiotensinogen, and αSMA) which may counteract some of the inhibitory effects on fibrosis. Furthermore, αSMA-positive cells were not reduced in the tissue. This might explain why nintedanib is less effective clinically than might be predicted from *in vivo* animal experiments and studies on lung cells.

Pirfenidone exhibited only weak anti-fibrotic activity in this model at the level of gene expression, and did not inhibit αSMA expression at the gene level or the protein level in the tissue. However, pirfenidone did not increase the gene expression of any pro-fibrotic molecules, and thus while exhibiting potentially less anti-fibrotic activity than nintedanib in human lung tissue, it did not counteract these inhibitory effects, which may explain why it is as effective as nintedanib clinically (King et al., 2014; Richeldi et al., 2014).

Fibroblast numbers and αSMA-positive cells were increased significantly in human lung tissue following 7 days of TGFβ1 treatment. There were more αSMA-positive cells than fibroblast-specific protein-positive cells indicating that not all αSMA-positive cells are (myo) fibroblasts, in keeping with the known expression of αSMA by other cell types. The reduction in fibroblast-specific protein-positive cells with both pirfenidone and nintedanib would be in keeping with the inhibition of acutely proliferating fibroblasts within the tissue, which is consistent with the ability of these drugs to inhibit the proliferation of human lung fibroblasts in culture (Conte et al., 2014; Hostettler et al., 2014). However, the failure to inhibit αSMA mRNA or protein expression in the tissue suggests that cells transitioning to myofibroblasts are not inhibited. Some of these cells could potentially be epithelial cells undergoing epithelial-mesenchymal-transition which is well recognised in IPF (Willis et al., 2005). These data on αSMA expression conflict with animal models of fibrosis, which suggest that pirfenidone and nintedanib decrease myofibroblast accumulation and differentiation, but are in keeping with the most recent histopathological results from IPF patients treated with either nintedanib and pirfenidone, where both drugs failed to reduce the density of the pathognomonic fibroblast foci (Zhang et al., 2019).

Understanding the potential anti-fibrotic effects of nintedanib and pirfenidone in TGFβ1-stimulated human lung tissue provide a potential benchmark with which to compare novel putative anti-fibrotic compounds. K_Ca_3.1 ion channels are implicated in fibrosis in several organs, and demonstrate a major role in pro-fibrotic human lung (myo) fibroblast activities (Roach and Bradding, 2020), and in a sheep model of bleomycin-dependent lung fibrosis (Organ et al., 2017). We previously described the anti-fibrotic effects of the K_Ca_3.1 ion channel in our *ex vivo* human lung model of fibrogenesis and showed significant inhibition of 28 TGFβ1-dependent genes including many molecules implicated in IPF pathophysiology, as well as reduced tissue fibroblast numbers, αSMA protein expression and tissue type I and III collagen expression. There was no upregulation of pro-fibrotic gene expression. We have further explored the molecular pathways that are likely to be impacted by nintedanib, pirfenidone and senicapoc, such as ECM-related molecules, cytokines and chemokines, growth factors, TGFβ superfamily members, and transcription factors (Supplementary Figures 3, 4, 5). Senicapoc inhibited molecules within all of these pathways, whereas nintedanib was more restricted and pirfenidone only inhibited MMPs within the ECM remodelling pathway. Coupled with the factor analysis of co-regulated genes, where senicapoc shows greater activity than either nintedanib or pirfenidone, and the potentially pro-fibrotic activity of nintedanib, we would predict that senicapoc will be more effective at treating human lung fibrosis than the two currently licensed drugs. Senicapoc was well tolerated in phase 3 trials of sickle cell disease over 12 months (Ataga et al., 2011), and there is clearly a strong case for progressing to clinical trials in human IPF. We expect that benchmarking against nintedanib and pirfenidone will be useful in predicting the clinical efficacy of other novel anti-fibrotic compounds.

Lipoxin A4 and a stable analogue have demonstrated anti-fibrotic activity in HLMFs and the mouse bleomycin lung fibrosis model (Martins et al., 2009; Roach et al., 2015a). There was no effect of lipoxin A4 in this *ex vivo* human lung model, which suggests that lipoxin A4 is unlikely to demonstrate efficacy in human IPF. It may simply not be stable enough in this model to exert anti-fibrotic activity, even with daily dosing as used here, and it remains possible that stable analogs might be more effective.

No model of disease, animal or human will be perfect, but if a model can be shown to reliably predict drug efficacy in IPF this would be a major advance. Our model does not develop over years unlike clinical IPF, and does not allow for the recruitment of cells from the blood such as fibrocytes. While the gene expression changes in our model are very similar to those reported in IPF tissue, CXCR4 for example is down regulated and so this model would not be suitable for studying CXCR4-related pathways. Further approaches can also shed light on drug mechanism of action in IPF, such as the work of Kwapiszewska et al. (Kwapiszewska et al., 2018). They used transcriptomic analysis to examine the molecular changes in the fibroblasts and lungs of people with IPF who had progressed to lung transplant, and demonstrated alteration of several pathways linked to extracellular matrix architecture and inflammation in patients receiving pirfenidone compared to those who had not received it. Explanted IPF tissue like this is in short supply and therefore difficult to obtain and work with, and the fact that these patients had progressed to lung transplantation shows that pirfenidone was of limited benefit, and this approach is not practical for assessing drugs at the pre-clinical development stage. Studying the effects of drugs in IPF tissue *ex vivo* is also potentially useful (Mercer et al., 2016) but again limited by tissue availability, and it is unclear whether explanted severely fibrotic tissue is too far down the fibrotic pathways to provide useful information on drugs that might halt disease progression. Clinical trials will ultimately prove whether our model described here is better than other approaches used to-date.

In summary, we have provided novel data regarding the molecular effects of nintedanib and pirfenidone in human lung tissue displaying early features of fibrosis after exposure to TGFβ1. Both drugs demonstrated some anti-fibrotic activity, but the effects of both drugs on gene expression were modest, and nintedanib also upregulated some pro-fibrotic molecules. This may explain why these molecules are only partially effective at treating human IPF, and suggests that there is substantial room for improvement with the development of new compounds. Towards that goal, the K_Ca_3.1 channel blocker senicapoc holds great promise when benchmarked against both nintedanib and pirfenidone.

## Data Availability

The original contributions presented in the study are included in the article/Supplementary Material, further inquiries can be directed to the corresponding author.
