# Supplementary material for: Evaluation of Pirfenidone and Nintedanib in a Human Lung Model of Fibrogenesis
Source: Front Pharmacol. 2021 Oct 12;12:679388. doi: 10.3389/fphar.2021.679388 (PMC8546112; doi:10.3389/fphar.2021.679388)
Supplement: Supplementary file 1 [file DataSheet1.docx]

Supplement Material


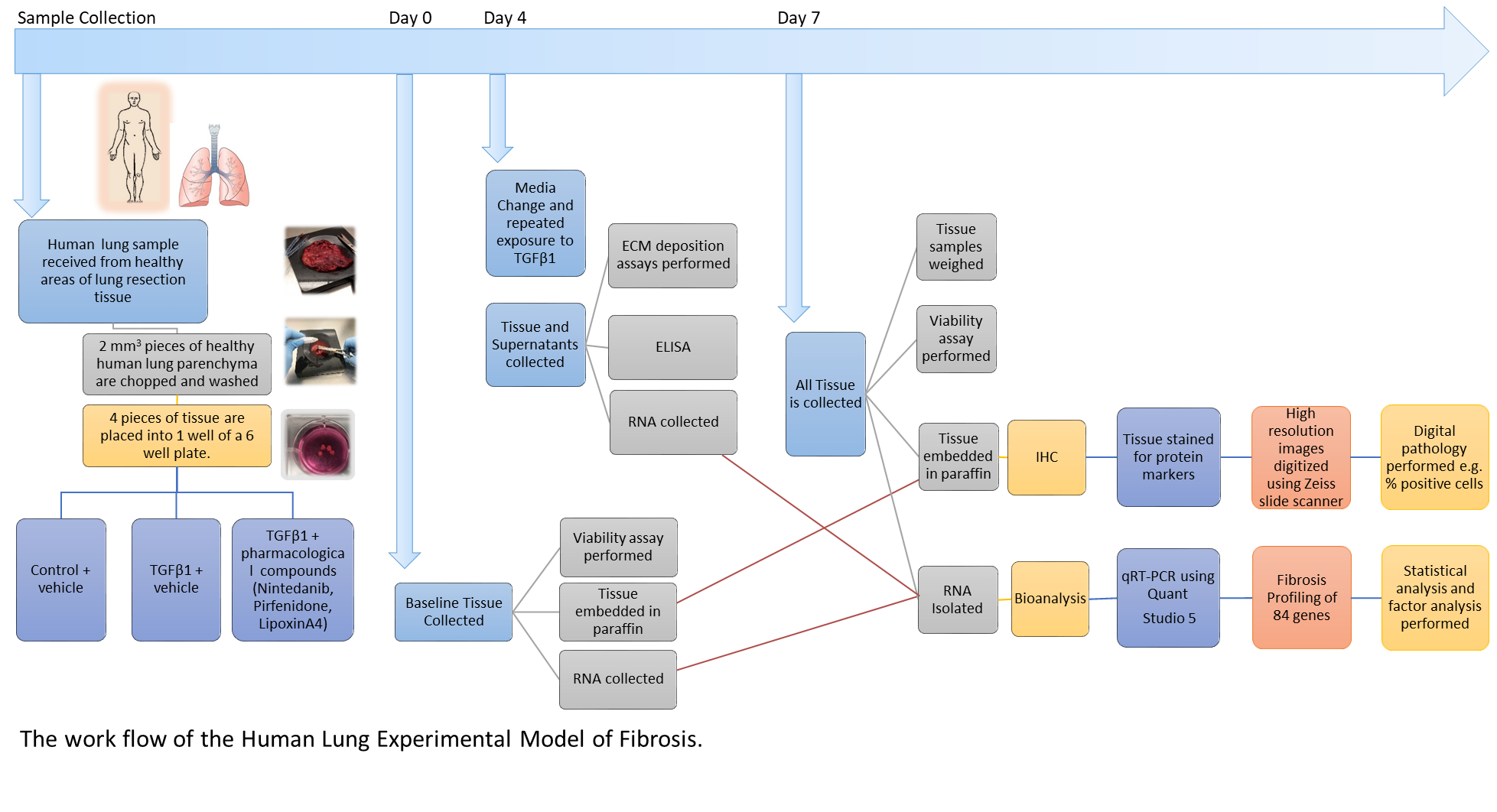
Supplementary Figure 1.

# Supplementary Table 1.

List of gene specific primer pairs of senescence markers

| Gene | Primer sequence (5’- 3’) | Harvard Primer bank ID: |
| --- | --- | --- |
| p16 | F: 5’ GATCCAGGTGGGTAGAAGGTC 3’  R: 5’ CCCCTGCAAACTTCGTCCT 3’ | 17738298a1 |
| p53 | F: 5’ CAGCACATGACGGAGGTTGT 3’  R: 5’ TCATCCAAATACTCCACACGC 3’ | 371502118c1 |
| PLD3 | F: 5’ AAGCCTAAACTGATGTACCAGGA 3’  R: 5’ GCCTCAATCTCATTCATGGGC 3’ | 166197682c1 |
| B2M | F: 5’ GAGGCTATCCAGCGTACTCCA 3’  R: 5’ CGGCAGGCATACTCATCTTTT 3’ | 166197682c1 |
| EBP50 | F: 5’ GGCTGGCAACGAAAATGAGC 3’  R: 5’ TGTCGCTGTGCAGGTTGAAG 3’ | 381214354c1 |
| VPS26A | F: 5’ TTCAGGAAAGGTAAACCTAGCCT 3’  R: 5’ ATTGGCACCGATGTAAGATTCAT 3’ | 78482612c1 |
| IL-6 | F: 5’ ACTCACCTCTTCAGAACGAATTG 3’  R: 5’ CCATCTTTGGAAGGTTCAGGTTG 3’ | 224831235c1 |
| IL-1B | F: 5’ ATGATGGCTTATTACAGTGGCAA 3’  R: 5’ GTCGGAGATTCGTAGCTGGA 3’ | 27894305c1 |
| DEP1 | F: 5’ AGTACACACGGCCCAGCAAT 3’  R: 5’ GAGGCGTCATCAAAGTTCTGC 3’ | N/A |
| ARMCX3 | F: 5’ TCTGGGGCCAGGTATAATGAC 3’  R: 5’ GGAAGCCCGTTTCTGGACA 3’ | 47578120c1 |
| GAPDH | F: 5’ TCTCTGCTCCTCCTGTTC 3’  R: 5’ GCCCAATACGACCAAATCC 3’ | N/A |

# Table 2.

List of Antibodies, manufacturers and dilutions used for immunohistochemistry.

| Antibody | Manufacturer | Product code | Antigen retrieval | Stock Concentration | Dilution |
| --- | --- | --- | --- | --- | --- |
| SMA (clone 1A4) | Dako | IR611 | High | RTU | RTU |
| Fibroblast surface protein | Sigma | F4771 | Low | 0.2mg/ml | 1/500 |
| Vimentin | Dako | M0725 | Low | 156mg/ml | 1/5000 |
| Collagen I | Abcam | ab34710 | Low | 1mg/ml | 1/500 |
| Collagen III | Sigma | C7805 | Low | 3.3mg/ml | TBC |
| Mast cell tryptase | Dako | M7052 | High | RTU | RTU |
| CD68 | Dako | IR609 | High | RTU | RTU |
| Caspase 3 | Sigma | HPA002643 | Low | 0.05mg/ml | 1/200 |
| IgG2a | Dako | X0943 | High | 100mg/L | - |
| IgG1 | Dako | X0931 | Low | 100mg/L | - |
| IgGRabbit | Immunostep | RBPLPU-01mg | Low | 100µg/ml | - |
| IgM | Sigma | M5909 | Low | 200ug/ml | - |

* Ready to use (RTU)


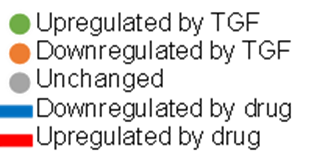

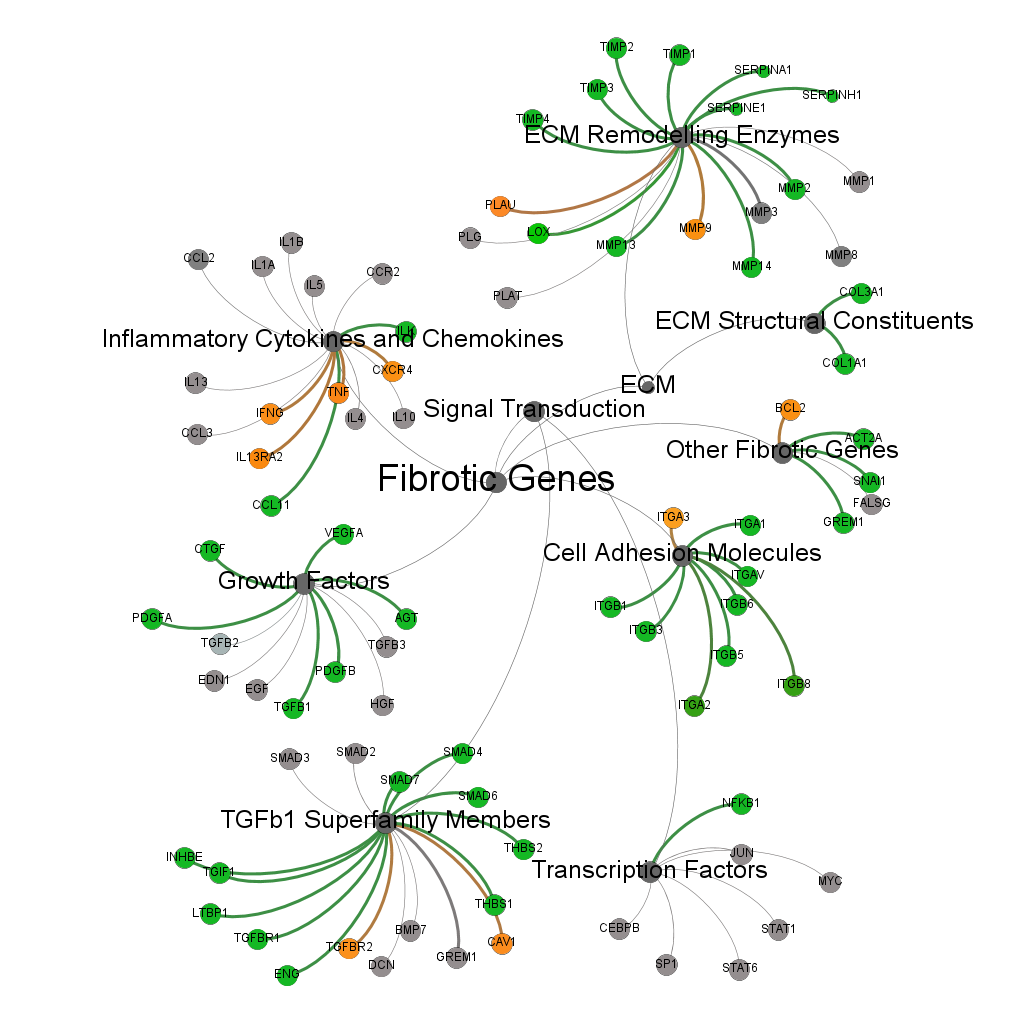
Supplementary Figure 2

TGFβ1-stmulated


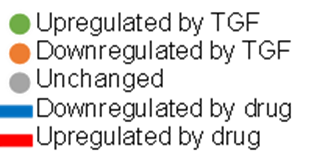

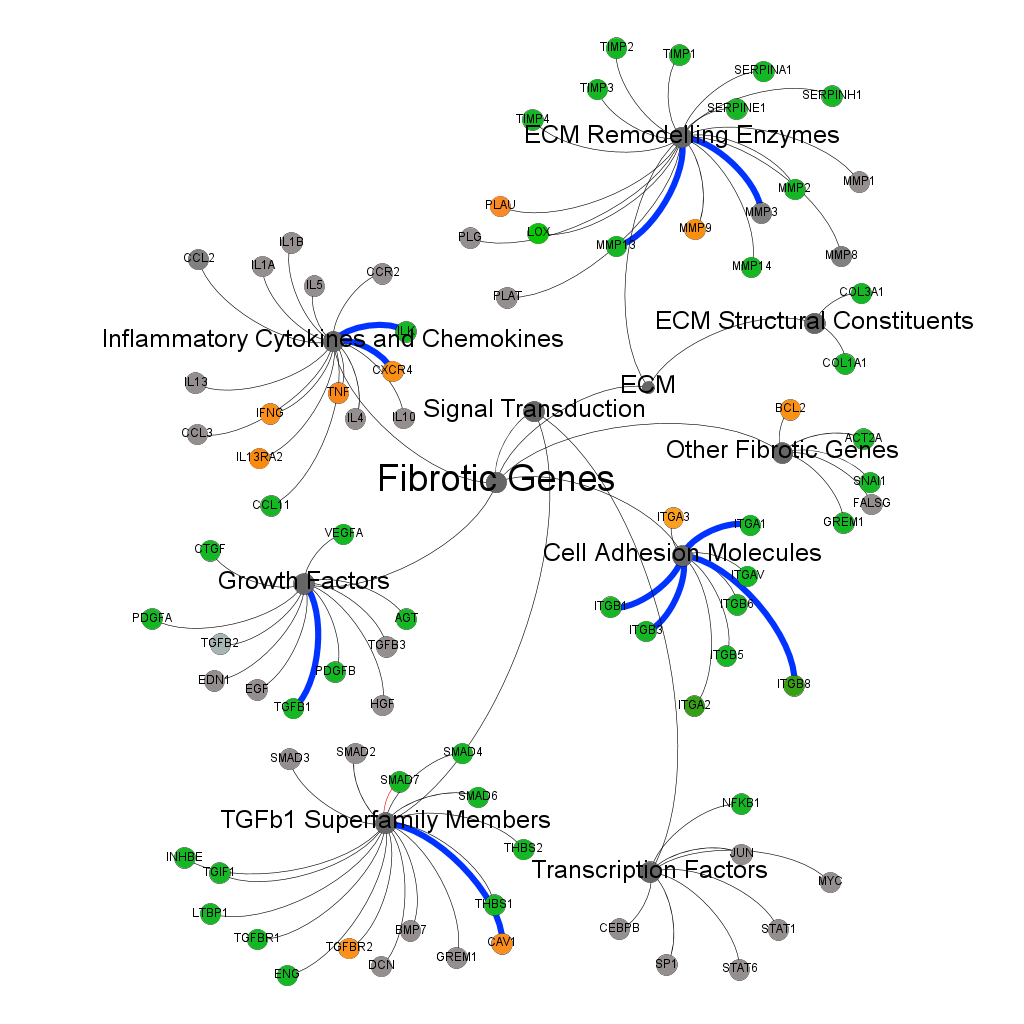
Supplementary Figure 3

Pirfenidone

Supplementary Figure
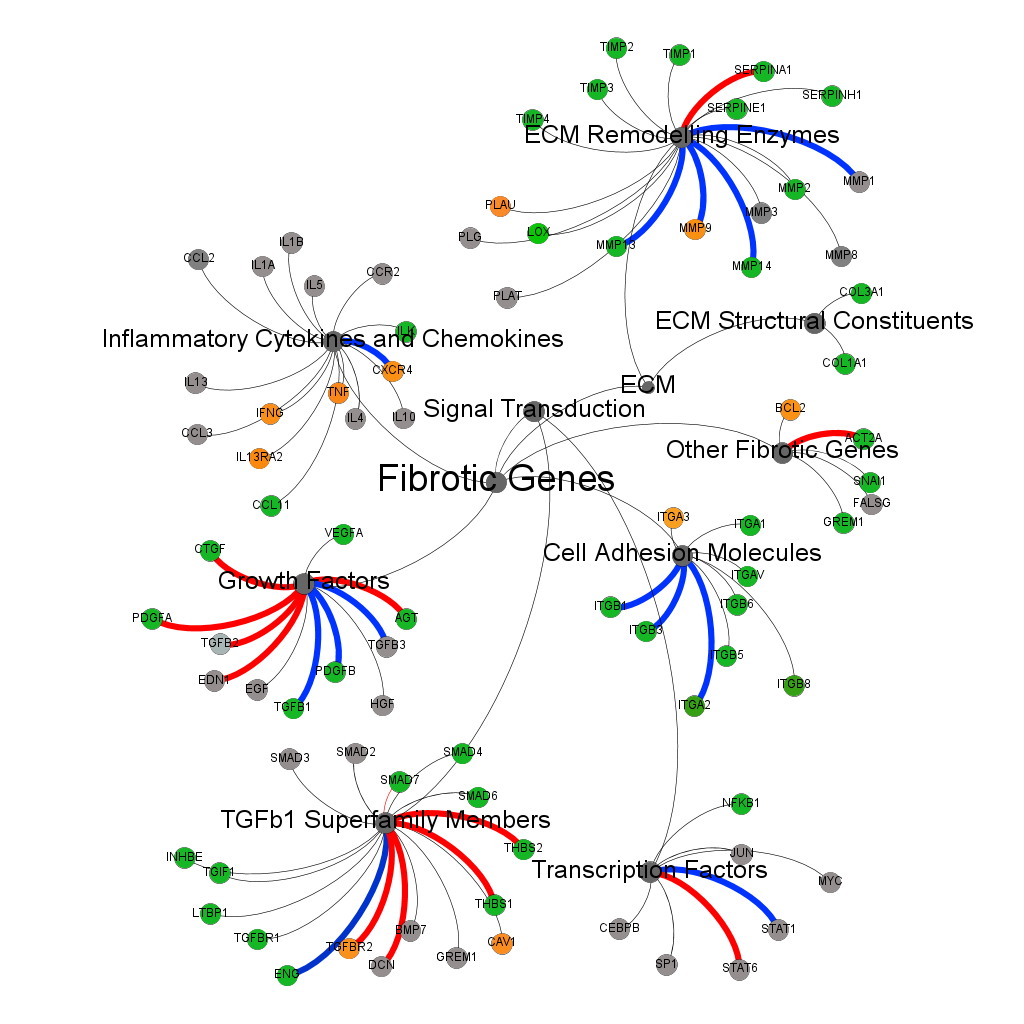
4

Nintedanib


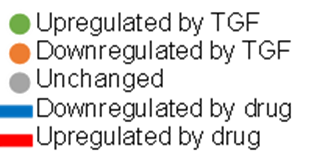


Supplementary Figure
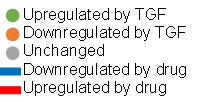

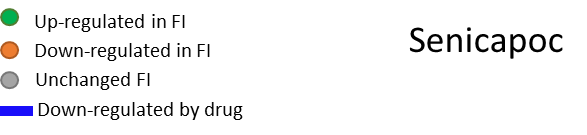

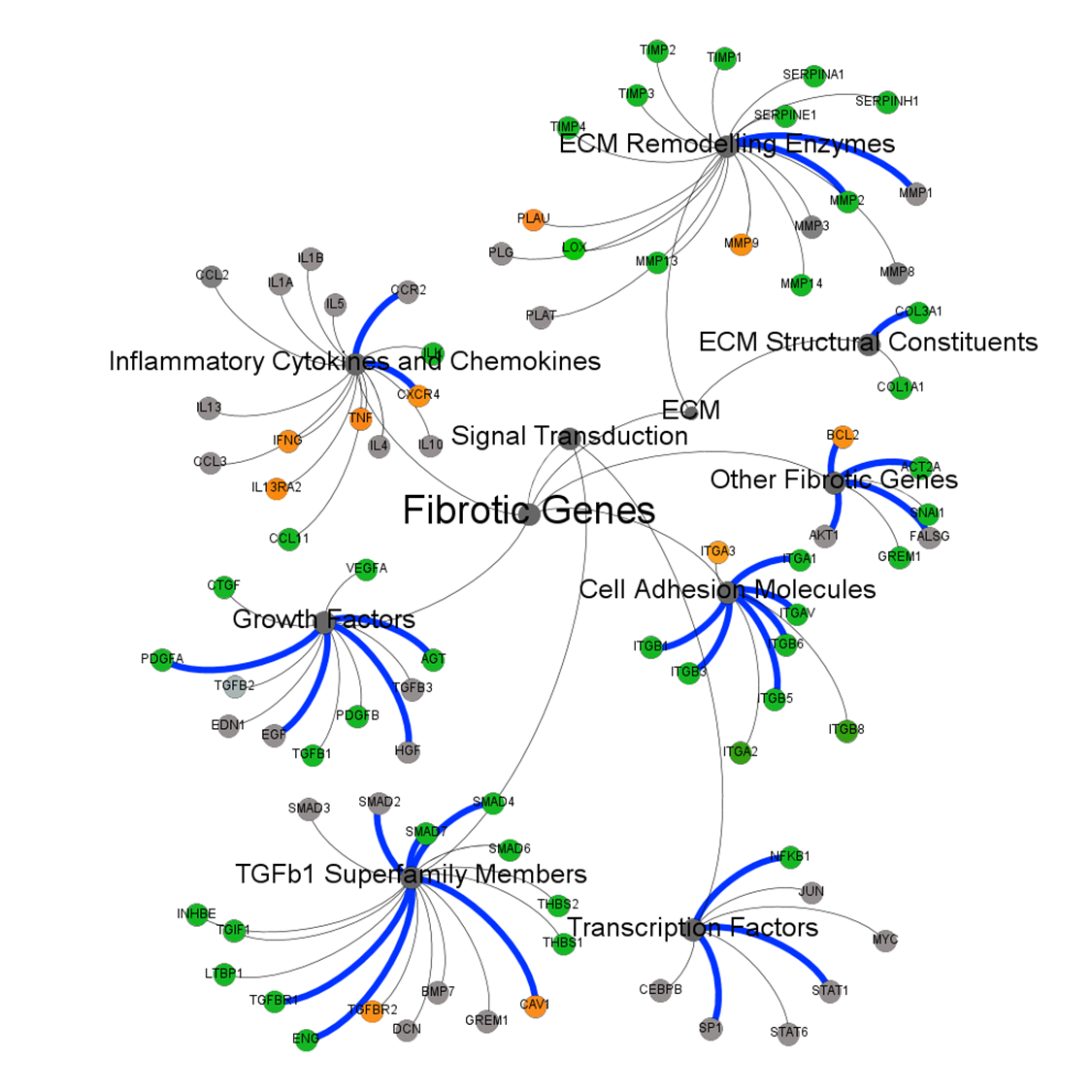
5

Supplementary Figure 6

The percentage of cells positive for the macrophage marker CD68 showed no significant changes in total, airspace or resident tissue macrophages numbers following treatment with nintedanib (1 µM, n=9) or pirfenidone (500 µM, n=11).

Total macrophages

Airspace macrophages

Tissue macrophages

Supplementary Figure 7

TGF vs TGF+Lipoxin

NS vs TGF

Lipoxin A4 10 nM with daily dosing
